# Clinical significance of MYC family protein expression in surgically resected high‐grade neuroendocrine carcinoma of the lung

**DOI:** 10.1111/1759-7714.14804

**Published:** 2023-01-24

**Authors:** Yuki Oshima, Tomohiro Haruki, Shinji Matsui, Karen Makishima, Tomohiko Sakabe, Yoshihisa Umekita, Hiroshige Nakamura

**Affiliations:** ^1^ Department of Surgery, Division of General Thoracic Surgery Faculty of Medicine, Tottori University Yonago Japan; ^2^ Department of Pathology, Division of Organ Pathology Faculty of Medicine, Tottori University Yonago Japan

**Keywords:** immunohistochemistry, MYC, neuroendocrine carcinoma, prognostic factor

## Abstract

**Objectives:**

MYC family genes including *MYC*, *MYCN*, and *MYCL* are amplified and overexpressed as oncogenic drivers in high‐grade neuroendocrine carcinoma of the lung (HGNEC), but little is known about their clinical significance. This study evaluated the prognostic impact of MYC family protein expression in patients with surgically resected HGNEC.

**Methods:**

Immunohistochemical analyses were performed on 83 resected specimens of HGNEC using antibodies against MYC family proteins (c‐MYC, n‐MYC, and l‐MYC). When nuclear staining of any intensity in ≥10% of tumor cells showed immunoreactivity with any one or more of c‐MYC, n‐MYC, or l‐MYC, the specimens were defined as MYC family‐positive.

**Results:**

A total of 83 patients were analyzed. MYC family‐positive status was observed in 33.7% (28 of 83 cases) and was not correlated with clinicopathological factors. The protein expression was mutually exclusive and no duplicate cases were observed. A log‐rank test showed that MYC family‐positive status was significantly associated with shorter overall survival (OS) (*p* = 0.003) and recurrence‐free survival (RFS) (*p* = 0.039). According to Cox multivariate analysis, MYC family‐positive status had a significant effect on shorter OS (hazard ratio [HR] = 2.217, 95% confidence interval [CI] 1.179–4.169, *p* = 0.014) and RFS (HR = 1.802, 95% CI 1.014–3.202, *p* = 0.045). In patients with pathological stage I, MYC family‐positive status also showed significantly poor OS (HR = 2.847, 95% CI 1.236–6.557, *p* = 0.014) and RFS (HR = 2.088, 95% CI 1.006–4.332, *p* = 0.048) in the multivariate analysis.

**Conclusions:**

MYC family protein expression could be an independent unfavorable prognostic factor in patients with surgically resected HGNEC.

## Graphical abstract

We demonstrated the prognostic impact of MYC family protein expression in patients with surgically resected high‐grade neuroendocrine carcinoma (HGNEC) of the lung. MYC family protein expression was an independent poor prognostic factor for 5‐year overall survival and recurrence‐free survival. MYC family protein expression could be a useful indicator for assessing the aggressive clinical behavior of HGNEC.

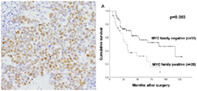

## INTRODUCTION

Lung cancer was the second most diagnosed cancer and the leading cause of cancer death worldwide in 2020, with an estimated 2.2 million new cancer cases and 1.8 million deaths.^1^ high‐grade neuroendocrine carcinoma (HGNEC) comprises small‐cell lung carcinoma (SCLC) and large‐cell neuroendocrine carcinoma (LCNEC), with SCLC and LCNEC accounting for 15% and 3% of all lung carcinomas, respectively.^2^ HGNEC has a highly aggressive clinical course even in early‐stage patients who have undergone surgical resection.^3^, ^4^ While targeted therapy and immunotherapy have been developed, especially in lung adenocarcinoma,^5^ the landscape of clinical treatment for HGNEC has not improved significantly in the last three decades. It is necessary to develop new criteria that can be used for assessing the risk of recurrence and death in patients with HGNEC. Moreover, identifying therapeutic targets and establishing new therapies is an urgent issue.

The MYC family genes consist of *MYC*, *MYCN*, and *MYCL*, all of which belong to the superfamily of basic helix‐loop‐helix leucine zipper transcription regulators.^6^, ^7^, ^8^ MYC family proteins have essentially the same multidomain‐type structure and play important roles in development, differentiation, cell proliferation, cell death, and stem cell self‐renewal.^7^, ^8^, ^9^ It has been reported that MYC family protein expression is a significant prognostic factor in various human tumors.^10^, ^11^, ^12^ Survival of patients with MYC family‐driven neuroblastomas in which tumors were n‐MYC‐ and/or c‐MYC‐positive was significantly worse than that of patients without non‐MYC family‐driven tumors.^10^ Chen et al. reported that l‐MYC protein expression was associated with significantly unfavorable outcomes of gastric cancer.^11^ However, colorectal cancer with c‐MYC overexpression demonstrated improved 5‐year survival compared with the c‐MYC‐negative group.^12^

With respect to HGNEC, a small number of studies have investigated the correlation between the protein expression of MYC family members and clinicopathological features,^13^, ^14^ but little is known about the prognostic impact. In particular, because of its low incidence and the rarity of surgical treatment, very few reports used sufficient tumor specimens. In this study, we analyzed the protein expression of MYC family members by immunohistochemistry and evaluated the prognostic significance in patients with surgically resected HGNEC.

## METHODS

### Patients and tumor specimens

We collected 103 tissue samples of HGNEC surgically resected at Tottori University Hospital and four other affiliated hospitals (Tottori Prefectural Central Hospital, Tottori Prefectural Kousei Hospital, Yonago Medical Center, and Matsue Medical Center) between January 2005 and December 2019. Twenty patients were excluded because of incomplete resection (*n* = 18) and insufficient tissue remaining (*n* = 2), therefore 83 cases with HGNEC were enrolled in our study. Pathological diagnosis was performed according to the criteria of the current World Health Organization classification^2^ and the 8th edition of the TNM classification of lung cancer.^15^ LCNEC was defined as non‐small‐cell carcinoma with neuroendocrine morphology (organoid nesting, palisading, rosettes, and trabeculae), high mitotic count (>10 mitoses/2 mm^2^), and positive immunohistochemical staining for one or more neuroendocrine markers (chromogranin A, synaptophysin, and CD56).^2^ The patients' clinicopathological data were obtained from medical records. This study was approved by the ethics committee of the Faculty of Medicine, Tottori University in May 2020 (20A002) and by the certified review board of each participating institution.

### Immunohistochemistry

Formalin‐fixed and paraffin‐embedded specimens were cut into 4‐μm thick specimens. After deparaffinization and rehydration, the specimens were pretreated in citrate buffer (0.01 M, pH 6.0) for 30 min in a water bath at 95°C for c‐MYC or in a decloaking chamber (NxGen; Biocare Medical) for n‐MYC and l‐MYC. Sections were incubated with 3% H_2_O_2_ in absolute methanol for 30 min to block endogenous peroxidase activity. Nonspecific binding was blocked using blocking buffer (BLOCK ACE; Megmilk Snow Brand). The sections were then incubated at 4°C overnight with anti‐c‐MYC antibody (dilution 1:200, ab32072; Abcam), anti‐n‐MYC antibody (dilution 1:600, #51705; Cell Signaling Technology), or anti‐l‐MYC antibody (dilution 1:200, #PA5‐41114; Invitrogen). Subsequently, they were incubated with EnVision+ System HRP (Dako Agilent Technologies). Finally, the slides were incubated with diaminobenzidine (DAB) solution (liquid DAB + substrate, imidazole‐HCI buffer, pH 7.5, containing hydrogen peroxide and an antimicrobial agent; Dako) and counterstained with hematoxylin.

### Evaluation of immunohistochemical findings

Nuclear staining of any intensity in ≥10% of tumor cells was defined as positive based on the published papers.^11^, ^12^ All other patterns were classified as negative. When nuclear staining of any intensity in ≥10% of tumor cells showed immunoreactivity with any one or more of c‐MYC, n‐MYC, or l‐MYC, the specimens were defined as MYC family‐positive. All slides were evaluated independently by Y.O. and K.M., who were blinded to the patients' clinicopathological data. When different interpretations were obtained, we reviewed the slides until a consensus was obtained.

### Statistical analysis

All statistical analyses were performed using SPSS version 27 (IBM SPSS Statistics; IBM Corporation). The association between MYC family protein expression status and clinicopathological factors was evaluated by nonparametric tests. The chi‐squared test was used when there were two categorical variables of interest and the Kruskal–Wallis test was used when there were three or more variables. For the survival analysis, we estimated the overall survival (OS) and recurrence‐free survival (RFS). OS was defined as the period from surgery to the date of death from any cause. RFS was defined as the period from the date of surgery to the date of recurrence or death from any cause. The cases of the patients who were alive were censored at the time of their last follow‐up visit. Survival curves were computed according to the Kaplan–Meier method and differences in OS and RFS were analyzed using the log‐rank test. The Cox regression hazard model was used to evaluate the effect of various factors on OS and RFS to observe the independent prognostic value of MYC family protein expression status. All tests were two‐sided, and *p* < 0.05 was considered to be statistically significant in all tests.

## RESULTS

### Patient characteristics

The clinicopathological characteristics of the 83 cases with HGNEC are summarized in Table 1. The median age was 72 (range 50–90) years, 72 (86.7%) patients were male, and 75 were heavy smokers (≥30 pack‐years). Surgical procedures included lobectomy (69.9%), segmentectomy (4.8%), wedge resection (20.5%), bilobectomy (3.6%), and pneumonectomy (1.2%). The histological diagnosis was SCLC in 36 (43.4%), LCNEC in 27 (32.5%), combined SCLC in 6 (7.2%), and combined LCNEC in 14 (16.9%). The pathological stage was stage I in 56 (67.5%), stage II in 17 (20.5%), and stage III in 10 (12.0%). Adjuvant chemotherapy was administered to 49 (59.0%) patients. Among them, 41 patients received cisplatin or carboplatin with etoposide.

**TABLE 1:** Clinicopathological characteristics of patients

Factors	Numbers	Percentage (%)
Age (median, range)	72 (50–90)	
Sex		
Male	72	86.7
Female	11	13.3
Smoking status		
<30 pack‐years	8	9.6
30–60 pack‐years	46	55.4
>60 pack‐years	29	34.9
Respiratory comorbidities		
Yes	33	39.8
No	50	60.2
Cardiovascular comorbidities		
Yes	19	22.9
No	64	77.1
Surgical procedure		
Lobectomy	58	69.9
Segmentectomy	4	4.8
Wedge resection	17	20.5
Bilobectomy	3	3.6
Pneumonectomy	1	1.2
Lymph node dissection		
ND2	49	59.0
ND1	17	20.5
ND0	17	20.5
Histology		
SCLC	36	43.4
LCNEC	27	32.5
Combined SCLC	6	7.2
Combined LCNEC	14	16.9
Pathological stage		
I	56	67.5
II	17	20.5
III	10	12.0
Adjuvant chemotherapy		
Yes	49	59.0
No	34	41.0

*Abbreviations*: LCNEC, large‐cell neuroendocrine carcinoma; ND2, Mediastinal and hilar lymph node dissection; ND1, Hilar lymph node dissection; ND0, No lymph node dissection; SCLC, small‐cell lung carcinoma.

### Immunohistochemistry

**FIGURE 1:**
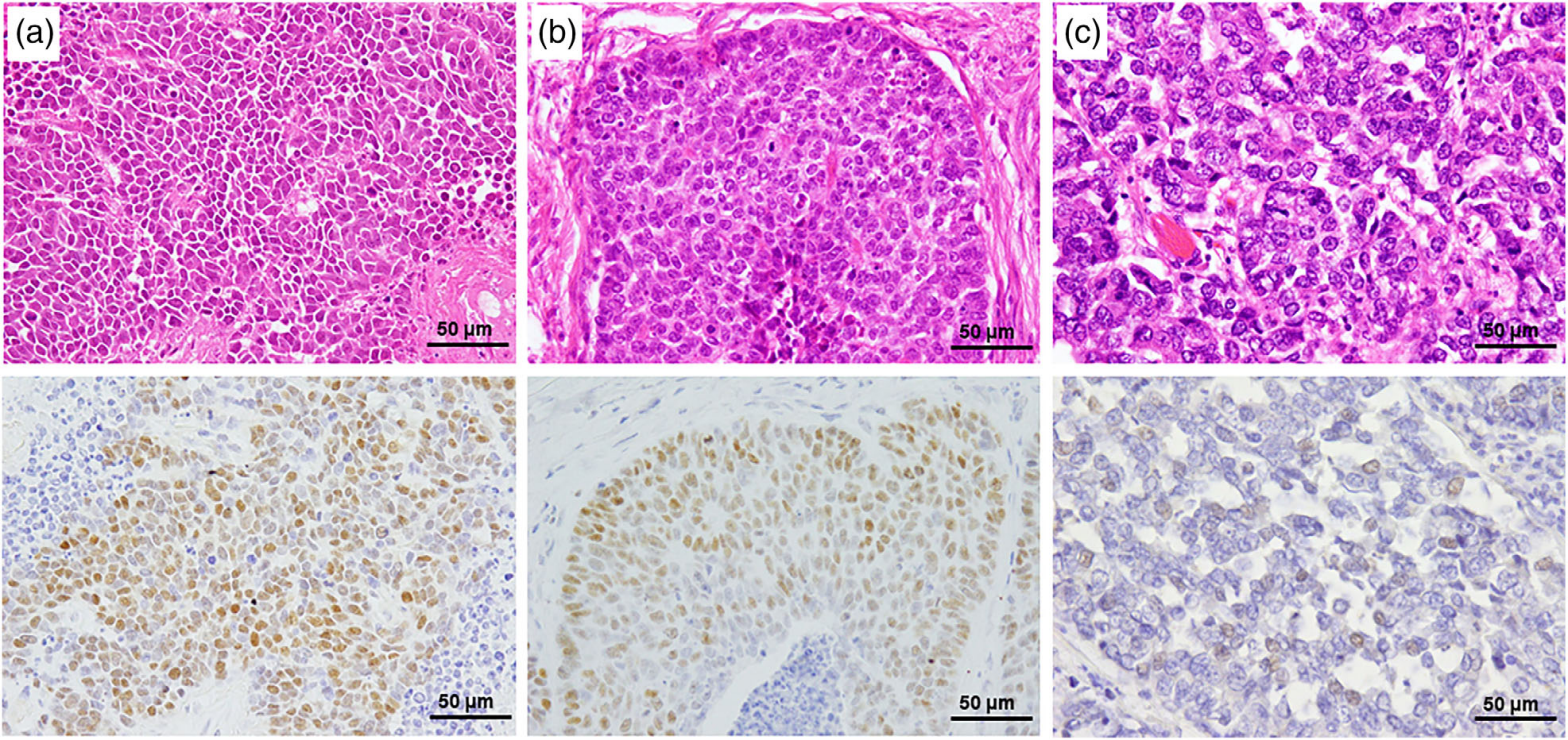
Representative images of high‐grade neuroendocrine carcinoma of the lung. (a) Hematoxylin and eosin staining (H&E) and c‐MYC immunohistochemistry (IHC) of small‐cell lung carcinoma. The nuclei of carcinoma were positive for c‐MYC. (b) H&E and n‐MYC IHC of large‐cell neuroendocrine carcinoma (LCNEC). The nuclei of carcinoma were positive for n‐MYC. (c) H&E and l‐MYC IHC of LCNEC. The nuclei of carcinoma were positive for l‐MYC. Original magnification is ×400 for H&E and IHC

Representative immunohistochemical staining of MYC family proteins is shown in Figure 1. Among 83 cases, the expression of c‐MYC, n‐MYC, and l‐MYC protein was detected in 22 (26.5%), 3 (3.6%), 3 (3.6%) specimens, respectively. The protein expression was mutually exclusive and no duplicate cases were observed. Supporting Information, Table S1 shows the correlation between MYC family protein expression status and histological type. The expression of c‐MYC protein was observed in all four histological types. There were no positive cases of n‐MYC in the combined types, and l‐MYC protein expression was found only in LCNEC or combined LCNEC.

### Correlation between clinicopathological characteristics and MYC family protein expression status

Table 2 shows a comparison of patient characteristics and MYC family protein expression. MYC family protein expression was not correlated with any clinicopathological factors.

**TABLE 2:** Association between MYC family protein expression and clinicopathological factors

Factors	Total (*n* = 83)	MYC family protein expression		
		Positive (*n* = 28)	Negative (*n* = 55)	*p* value
Age (year)				
<70	33	9	24	0.439
≥70	50	19	31	
Sex				
Male	72	25	47	0.743
Female	11	3	8	
Respiratory comorbidities				
Yes	33	8	25	0.212
No	50	20	30	
Cardiovascular comorbidities				
Yes	19	6	13	1
No	64	22	42	
Surgical procedure				
Lobectomy	58	19	39	0.811
Segmentectomy	4	1	3	
Wedge resection	17	6	11	
Bilobectomy	3	2	1	
Pneumonectomy	1	0	1	
Lymph node dissection				
ND2	49	14	35	0.377
ND1	17	8	9	
ND0	17	6	11	
Histology				
SCLC	36	11	25	0.534
LCNEC	27	12	15	
Combined SCLC	6	1	5	
Combined LCNEC	14	4	10	
Pathological stage				
I	56	16	40	0.311
II	17	7	10	
III	10	5	5	
Adjuvant chemotherapy				
Yes	49	16	33	0.818
No	34	12	22	

*Abbreviations*: LCNEC, large‐cell neuroendocrine carcinoma; ND2, Mediastinal and hilar lymph node dissection; ND1, Hilar lymph node dissection; ND0, No lymph node dissection; SCLC, small‐cell lung carcinoma.

### Survival analysis

**FIGURE 2:**
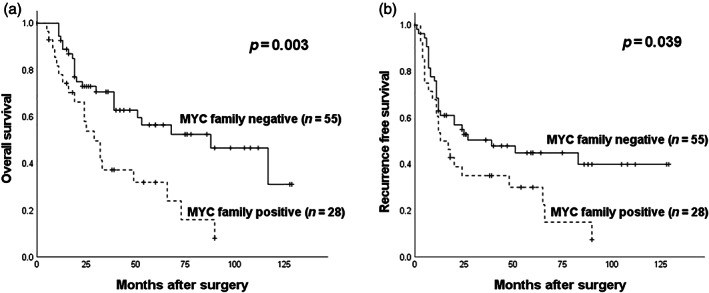
Kaplan–Meier survival curves for overall survival (a) and recurrence‐free survival (b) of 83 high‐grade neuroendocrine carcinoma of the lung based on MYC family protein expression status

The median follow‐up time was 30.0 months (range 0–129 months, mean 41.3 months). There were 46 (55.4%) recurrent cases out of 83 patients, which consisted of 33 distant, seven locoregional and distant, and six locoregional recurrences. Thirty‐five patients died of lung cancer progression and eight died of other causes. The survival curves of the patients are shown in Figure 2 and Supporting Information, Figure S1. The 5‐year OS rates of the c‐MYC‐positive and ‐negative groups were 37.6% and 51.8%, respectively, and the corresponding 5‐year RFS rates were 38.6% and 40.2%, respectively. The log‐rank test showed that the c‐MYC‐positive group had significantly shorter OS (*p* = 0.023) compared with the c‐MYC‐negative group (Supporting Information, Figure S1A,D). Of the three n‐MYC‐positive patients, all patients recurred and one died of cancer (Supporting Information, Figure S1B,E). Of the three l‐MYC‐positive patients, two patients died of cancer and one died of another cause (Supporting Information, Figure S1C,F). The 5‐year OS rates of MYC family‐positive and ‐negative groups were 31.9% and 56.5%, respectively, and the corresponding 5‐year RFS rates were 30.1% and 44.9%, respectively. The log‐rank test showed that the MYC family‐positive group had significantly shorter OS (*p* = 0.003) and RFS (*p* = 0.039) compared with the MYC family‐negative group (Figure 2). Univariate analysis showed a significant correlation between shorter OS and MYC family‐positive status (hazard ratio [HR] = 2.415, 95% confidence interval [CI] 1.314–4.439, *p* = 0.005) and adjuvant chemotherapy (HR = 2.125, 95% CI 1.163–3.881, *p* = 0.014). Multivariate analysis suggested that MYC family‐positive status (HR = 2.217, 95% CI 1.179–4.169, *p* = 0.014), sublobar resection (HR = 2.219, 95% CI 1.128–4.366, *p* = 0.021), pathological stage (HR = 2.213, 95% CI 1.101–4.445, *p* = 0.026), and adjuvant chemotherapy (HR = 2.218, 95% CI 1.207–4.075, *p* = 0.010) were independent prognostic factors for OS (Table 3). Univariate analysis showed a significant correlation between shorter RFS and MYC family‐positive status (HR = 1.764, 95% CI 1.012–3.074, *p* = 0.045), sublobar resection (HR = 2.019, 95% CI 1.161–3.511, *p* = 0.013), and adjuvant chemotherapy (HR = 1.921, 95% CI 1.100–3.355, *p* = 0.022). According to the multivariate analysis, MYC family‐positive status had a significant effect on RFS (HR = 1.802, 95% CI 1.014–3.202, *p* = 0.045) as well as adjuvant chemotherapy (HR = 1.968, 95% CI 1.120–3.461, *p* = 0.019) (Supporting Information, Table S2).

**TABLE 3:** Univariate and multivariate analysis of prognostic factors in all patients

Prognostic factors	Univariate			Multivariate		
	HR	95% CI	*p* value	HR	95% CI	*p* value
Age (year)	1.509	0.805–2.831	0.200			
≥70 vs. <70						
Sex	1.660	0.764–3.607	0.200			
Male vs. female						
Respiratory comorbidities	1.451	0.789–2.668	0.232			
Yes vs. no						
Cardiovascular comorbidities	0.774	0.370–1.620	0.497			
Yes vs. no						
Surgical procedure	1.780	0.975–3.250	0.061	2.219	1.128–4.366	0.021
Sublobar vs. lobectomy or more						
Histology	1.083	0.592–1.981	0.795			
SCLC or combined SCLC vs. others						
Pathological stage	1.817	0.988–3.342	0.055	2.213	1.101–4.445	0.026
II + III vs. I						
Adjuvant chemotherapy	2.125	1.163–3.881	0.014	2.218	1.207–4.075	0.010
None vs. performed						
MYC family protein expression	2.415	1.314–4.439	0.005	2.217	1.179–4.169	0.014
Positive vs. negative						

*Abbreviations*: CI, confidence interval; HR, hazard ratio; SCLC, small‐cell lung carcinoma.

To investigate the effects of MYC family protein expression and other clinicopathological factors on early‐stage HGNEC, we performed subset analyses for pathological stage I patients. The survival curves for pathological stage I patients are shown in Supporting Information, Figure S2.

The 5‐year OS rates of the MYC family‐positive and ‐negative groups were 40.9% and 66.6%, respectively, and the corresponding 5‐year RFS rates were 35.7% and 53.5%, respectively. The log‐rank test showed that the MYC family‐positive group had significantly shorter OS (*p* = 0.004) and RFS (*p* = 0.022). In the Cox multivariate model, MYC family protein expression was an independent poor prognostic factor for OS (*p* = 0.014) and RFS (*p* = 0.048) (Supporting Information, Tables S3 and S4).

## DISCUSSION

This study investigated the expression of MYC family proteins in surgically resected HGNEC and evaluated their contribution to survival in patients with this high‐grade tumor. The prognostic impact of the amplification and overexpression of the *MYC* gene family in HGNEC is not fully understood. In patients with untreated SCLC, Hwang et al. reported that *MYC* amplification and c‐MYC protein expression had no statistically significant effect on OS.^13^ Alves et al. showed that *MYC* amplification was associated with shorter survival time.^16^ In these reports, most cases were in the advanced stage and clinical information about treatment was not available. Qin et al. explored *MYCL1* amplification and l‐MYC and c‐MYC protein expression in 46 patients with surgically resected SCLC. Although they investigated clinicopathological characteristics and survival status, the number of positive cases was not sufficient to perform statistical analysis based on protein expression status.^14^ To the best of our knowledge, very few reports have focused on the prognostic impact of MYC protein expression in LCNEC of the lung.^17^ Amplification of *MYC* in SCLC cell lines is associated with increased susceptibility to Aurora kinase inhibitors.^18^, ^19^ Owonikoko et al. reported that alisertib/paclitaxel showed significant benefit over placebo/paclitaxel in resistant or refractory relapsed SCLC patients with c‐MYC protein expression.^20^ When considering the treatment strategy of HGNEC patients, it may be important to confirm the presence or absence of MYC protein expression in the future. We hypothesized that MYC family member protein expression would be a prognostic factor in patients with HGNEC and investigated the effects of clinicopathological features and MYC protein expression on prognosis in surgically resected cases for which a sufficient amount of tissue is available. As far as we know, this is the first report to investigate the correlation between the protein expression of MYC family members and clinicopathological features with detailed information such as surgical procedures and postoperative treatment.

The MYC family genes consist of *MYC*, *MYCN*, and *MYCL*. These three paralogs share structural and functional similarities,^21^, ^22^, ^23^ but the timing of expression and tissue specificity differ among them.^21^ c‐MYC is widely expressed in various tumors and ubiquitously expressed during histogenesis. n‐MYC is predominantly expressed in neural tissue and is deregulated in tumors such as neuroblastoma. l‐MYC is expressed in the lung and is particularly overexpressed in SCLC. The protein sequence of MYC contains several highly conserved segments, including the MYC homology box (MB), which regulate MYC function and are mostly shared by all three paralogs.^24^ Given the similarities between family members, we sought to investigate the clinical significance of protein expression not only for each paralog but also the ‘MYC family’. In our study, MYC family protein expression was an independent poor prognostic factor for 5‐year OS and RFS, not only in the entire cohort, but in pathological stage I according to the multivariate analysis. Notably, MYC family protein expression was an independent poor prognostic factor for OS and RFS, regardless of surgical procedure, pathological stage, or adjuvant chemotherapy. Our results were consistent with previous reports regarding the benefits of lobectomy and adjuvant chemotherapy in SCLC and LCNEC patients.^25^, ^26^, ^27^, ^28^ In addition, there was no significant difference in the 5‐year OS and RFS between patients with SCLC component and others. These results are similar to a large multicenter study of surgically resected neuroendocrine tumors of the lung, where the survival curves of LCNEC and SCLC were superimposed and there was no difference in survival.^29^ We speculated that MYC family protein expression may be a useful indicator for assessing the aggressive clinical behavior of HGNEC. For each MYC family member, c‐MYC protein expression was an independent prognostic factor for OS in patients with pathological stage I, but we failed to demonstrate statistical significance in the entire cohort. The main reason for our failure is probably the small number of cases and the shorter follow‐up period in this study. Moreover, the number of n‐MYC‐ and l‐MYC‐positive cases was too small to statistically evaluate the prognostic impact. The frequency of MYC family member protein expression varies from report to report.^14^, ^30^ Qin et al. reported the expression of c‐MYC and l‐MYC using surgically resected SCLC tissue, with positive rates of 8.7% and 6.5%, respectively.^14^ However, Qu et al. revealed that the expression of c‐MYC, n‐MYC, and l‐MYC protein was detected in 11.8%, 0.7%, and 31.9% of SCLC specimens, respectively.^30^ Factors contributing to this discordance may include differences in the antibodies used, immunohistochemistry protocols, positivity criteria, and the study population.

Several limitations should be mentioned when interpreting the results of the present study. First, this study was a retrospective, nonrandomized study with a limited sample size because of the rarity of the disease. However, we could evaluate statistical correlations between MYC family protein expression and prognosis by collecting specimens from participating institutions. Second, some clinical information, including tumor markers, postoperative complications, and performance status, was incomplete and could not be added to the analysis. Third, information about immunohistochemical staining other than MYC family members and neuroendocrine markers was incomplete. Rekhtman et al. reported that LCNEC consists of two distinct subtypes, SCLC‐like and NSCLC‐like subsets, by using next‐generation sequencing.^31^ In our study, immunohistochemical staining used to distinguish between these two subtypes, such as pRb, STK11, and Napsin A,^31^, ^32^ was not performed. Finally, this study may have been biased because management decisions such as surgical procedures, postoperative adjuvant therapy, and follow‐up intervals were left to each participating institution.

In conclusion, our study demonstrated the prevalence of MYC family protein expression and its unfavorable prognostic impact in patients with surgically resected HGNEC. MYC family protein expression may predict the prognosis of HGNEC and provides important information for the development of new therapeutic strategies in the future.

## AUTHORS CONTRIBUTIONS

All authors have made significant contributions to this paper. Conception and design: T.H., Y.O., and S.M. Data analysis and acquisition: T.H., Y.O., T.S, and K.M. Pathology: Y.U., Y.O., K.M., and S.M. Writing, review, and/or revision of the manuscript: Y.O. and T.H. Study supervision: H.N. and Y.U.

## CONFLICT OF INTEREST

The authors have no conflicts of interest to declare.

## Supporting information


**FIGURE S1.**Kaplan–Meier survival curves for overall survival (A–C) and recurrence‐free survival (D–F) of 83 high‐grade neuroendocrine carcinomas of the lung based on MYC family member (c‐MYC, n‐MYC, and l‐MYC) protein expression status
**FIGURE S2.** Kaplan–Meier survival curves for overall survival (A) and recurrence‐free survival (B) of 56 patients with pathological stage I based on MYC family protein expression status

**TABLE S1.** Correlation between MYC family protein expression status and histologic type
**TABLE S2.** Univariate and multivariate analysis of prognostic factors on recurrence‐free survival in all patients
**TABLE S3.** Univariate and multivariate analysis of prognostic factors on overall survival in patients with pathological stage I high‐grade neuroendocrine carcinoma of the lung
**TABLE S4.** Univariate and multivariate analysis of prognostic factors on recurrence‐free survival in patients with pathological stage I high‐grade neuroendocrine carcinoma of the lung
