# Supplementary figures and images for: Clinical significance of MYC family protein expression in surgically resected high‐grade neuroendocrine carcinoma of the lung
Source: Thorac Cancer. 2023 Jan 24;14(8):758–65. doi: 10.1111/1759-7714.14804 (PMC10008680; doi:10.1111/1759-7714.14804)

## Slide 1
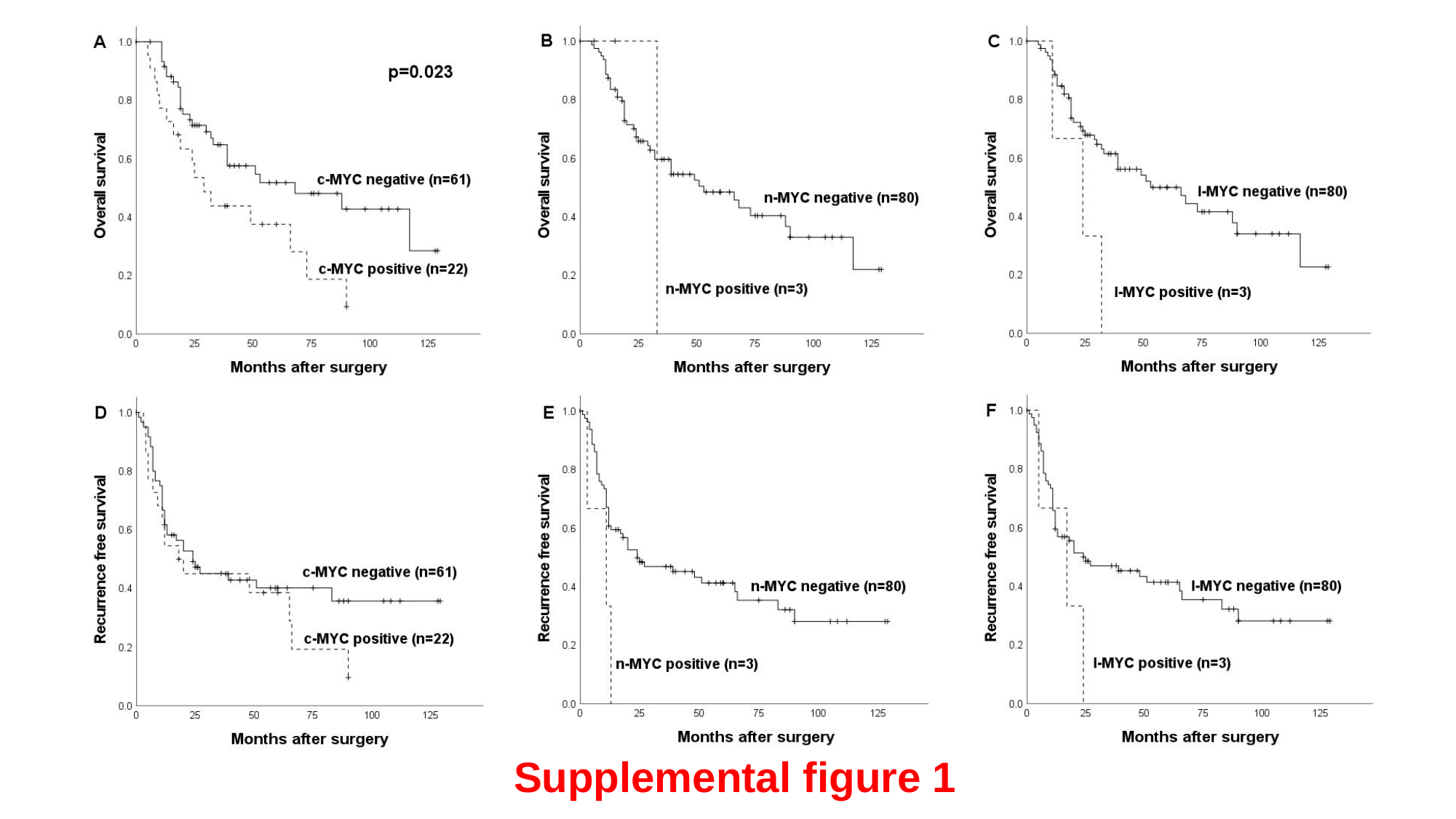

Supplemental figure 1

## Slide 2
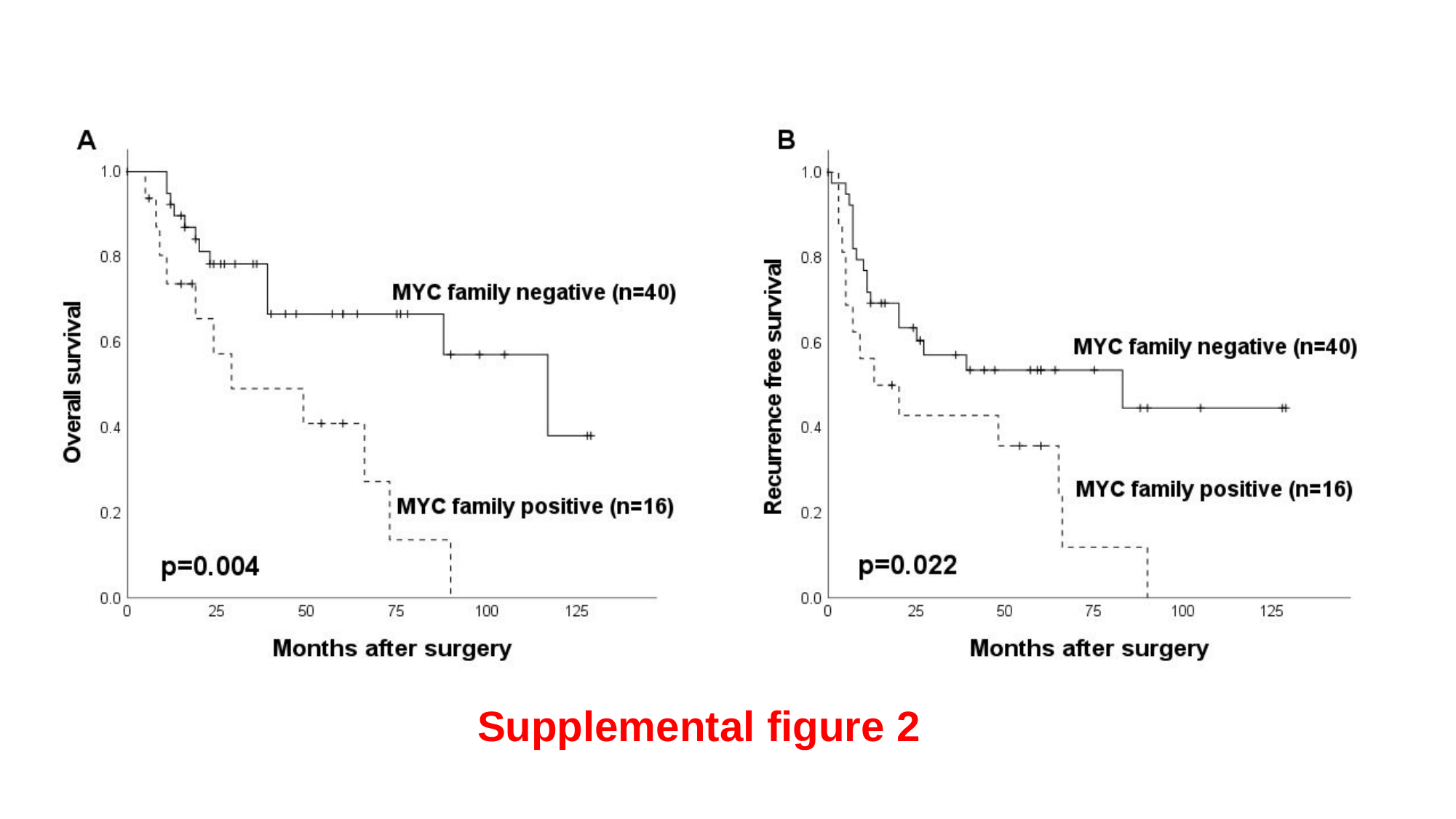

Supplemental figure 2

Supplement: Supplementary file 1 — FIGURE S1.Kaplan–Meier survival curves for overall survival (A–C) and recurrence‐free survival (D–F) of 83 high‐grade neuroendocrine carcinomas of the lung based on MYC family member (c‐MYC, n‐MYC, and l‐MYC) protein expression status FIGURE S2. Kaplan–Meier survival curves for overall survival (A) and recurrence‐free survival (B) of 56 patients with pathological stage I based on MYC family protein expression status [file TCA-14-758-s001.pptx]
