# Supplementary material for: Clinical significance of MYC family protein expression in surgically resected high‐grade neuroendocrine carcinoma of the lung
Source: Thorac Cancer. 2023 Jan 24;14(8):758–65. doi: 10.1111/1759-7714.14804 (PMC10008680; doi:10.1111/1759-7714.14804)
Supplement: Supplementary file 2 — TABLE S1. Correlation between MYC family protein expression status and histologic type TABLE S2. Univariate and multivariate analysis of prognostic factors on recurrence‐free survival in all patients TABLE S3. Univariate and multivariate analysis of prognostic factors on overall survival in patients with pathological stage I high‐grade neuroendocrine carcinoma of the lung TABLE S4. Univariate and multivariate analysis of prognostic factors on recurrence‐free survival in patients with pathological stage I high‐grade neuroendocrine carcinoma of the lung [file TCA-14-758-s002.docx]

**Supplemental table 1.** Correlation between MYC family protein expression status and histologic type

|  | c-MYC | |  | n-MYC | |  | l-MYC | |  | MYC family | |
| --- | --- | --- | --- | --- | --- | --- | --- | --- | --- | --- | --- |
|  | positive | negative |  | positive | negative |  | positive | negative |  | positive | negative |
| Histology | (n=22) | (n=61) |  | (n=3) | (n=80) |  | (n=3) | (n=80) |  | (n=28) | (n=55) |
| SCLC | 9 | 27 |  | 2 | 34 |  | 0 | 36 |  | 11 | 25 |
| Combined SCLC | 1 | 5 |  | 0 | 6 |  | 0 | 6 |  | 1 | 5 |
| LCNEC | 9 | 18 |  | 1 | 26 |  | 2 | 25 |  | 12 | 15 |
| Combined LCNEC | 3 | 11 |  | 0 | 14 |  | 1 | 13 |  | 4 | 10 |

**Supplemental table 2.**

Univariate and multivariate analysis of prognostic factors on recurrence-free survival in all patients

| Prognostic factors | Univariate | | |  | Multivariate | | |
| --- | --- | --- | --- | --- | --- | --- | --- |
|  | HR | 95% CI | *p*-value |  | HR | 95% CI | *p*-value |
| Age (year) ≥70 vs. <70 | 1.496 | 0.852 - 2.267 | 0.161 |  | 1.283 | 0.723 - 2.277 | 0.394 |
| Gender Male vs. Female | 1.358 | 0.636 - 2.898 | 0.430 |  |  |  |  |
| Respiratory comorbidities Yes vs. No | 1.603 | 0.922 - 2.788 | 0.095 |  | 1.693 | 0.952 - 3.012 | 0.073 |
| Cardiovascular comorbidities  Yes vs. No | 0.668 | 0.334 - 1.336 | 0.254 |  |  |  |  |
| Surgical procedure  Sublobar vs. Lobectomy or more | 2.019 | 1.161 - 3.511 | 0.013 |  | 1.717 | 0.979 - 3.011 | 0.059 |
| Histology  SCLC or Combined SCLC vs. Others | 1.108 | 0.638 - 1.923 | 0.716 |  |  |  |  |
| Pathological stage  II+III vs. I | 1.449 | 0.823 - 2.549 | 0.199 |  |  |  |  |
| Adjuvant chemotherapy  None vs. Performed | 1.921 | 1.100 - 3.355 | 0.022 |  | 1.968 | 1.120 - 3.461 | 0.019 |
| MYC family protein expression  Positive vs. Negative | 1.764 | 1.012 - 3.074 | 0.045 |  | 1.802 | 1.014 - 3.202 | 0.045 |

HR, Hazard ratio; CI, Confidence interval.

**Supplemental table 3.**

Univariate and multivariate analysis of prognostic factors on overall survival in patients with pathological stage I high-grade neuroendocrine carcinoma of the lung

| Prognostic factors | Univariate | | |  | Multivariate | | |
| --- | --- | --- | --- | --- | --- | --- | --- |
|  | HR | 95% CI | *p*-value |  | HR | 95% CI | *p*-value |
| Age (year) ≥70 vs. <70 | 1.395 | 0.596 - 3.266 | 0.443 |  |  |  |  |
| Gender Male vs. Female | 1.788 | 0.604 - 5.292 | 0.294 |  |  |  |  |
| Respiratory comorbidities Yes vs. No | 1.566 | 0.698 - 3.514 | 0.277 |  |  |  |  |
| Cardiovascular comorbidities  Yes vs. No | 0.712 | 0.242 - 2.100 | 0.539 |  |  |  |  |
| Surgical procedure  Sublobar vs. Lobectomy or more | 2.584 | 1.097 - 6.085 | 0.030 |  | 2.312 | 0.968 - 5.524 | 0.059 |
| Histology  SCLC or Combined SCLC vs. Others | 0.863 | 0.380 - 1.960 | 0.725 |  |  |  |  |
| T-factor  T2a vs. T1 | 2.184 | 0.948 - 5.034 | 0.067 |  |  |  |  |
| Pleural invasion  pl0 vs. pl1-3 | 1.546 | 0.690 - 3.467 | 0.290 |  |  |  |  |
| Lymphatic vessel invasion  Ly0 vs. Ly1-2 | 1.355 | 0.578 - 3.176 | 0.484 |  |  |  |  |
| Vascular invasion  V0 vs. V1-2 | 0.892 | 0.398 - 1.999 | 0.781 |  |  |  |  |
| Adjuvant chemotherapy  　None vs. Performed | 2.218 | 0.991 - 4.966 | 0.053 |  |  |  |  |
| MYC family protein expression  Positive vs. Negative | 3.157 | 1.385 - 7.194 | 0.006 |  | 2.847 | 1.236 - 6.557 | 0.014 |

HR: Hazard ratio. CI: Confidence interval.

**Supplemental table 4.**

Univariate and multivariate analysis of prognostic factors on recurrence-free survival in patients with pathological stage I high-grade neuroendocrine carcinoma of the lung

| Prognostic factors | Univariate | | |  | Multivariate | | |
| --- | --- | --- | --- | --- | --- | --- | --- |
|  | HR | 95% CI | *p*-value |  | HR | 95% CI | *p*-value |
| Age (year) ≥70 vs. <70 | 1.131 | 0.540 - 2.368 | 0.744 |  |  |  |  |
| Gender Male vs. Female | 1.170 | 0.408 - 3.359 | 0.770 |  |  |  |  |
| Respiratory comorbidities Yes vs. No | 1.778 | 0.869 - 3.636 | 0.115 |  |  |  |  |
| Cardiovascular comorbidities  Yes vs. No | 0.594 | 0.228 - 1.551 | 0.288 |  |  |  |  |
| Surgical procedure  Sublobar vs. Lobectomy or more | 2.907 | 1.360 - 6.216 | 0.006 |  | 2.477 | 1.149 - 5.342 | 0.021 |
| Histology  SCLC or Combined SCLC vs. Others | 1.080 | 0.532 - 2.194 | 0.831 |  |  |  |  |
| T-factor  T2a vs. T1 | 2.136 | 1.021 - 4.466 | 0.044 |  |  |  |  |
| Pleural invasion  pl0 vs. pl1-3 | 1.465 | 0.714 - 3.007 | 0.298 |  |  |  |  |
| Lymphatic vessel invasion  Ly0 vs. Ly1-2 | 1.310 | 0.627 - 2.737 | 0.474 |  |  |  |  |
| Vascular invasion  V0 vs. V1-2 | 0.954 | 0.470 - 1.936 | 0.895 |  |  |  |  |
| Adjuvant chemotherapy  None vs. Performed | 2.313 | 1.110 - 4.822 | 0.025 |  | 2.202 | 1.056 - 4.592 | 0.035 |
| MYC family protein expression  Positive vs. Negative | 2.235 | 1.092 - 4.574 | 0.028 |  | 2.088 | 1.006 - 4.332 | 0.048 |

HR: Hazard ratio. CI: Confidence interval.
